# P21 Hepatitis B-associated cryoglobulinemia and autoimmunity in the absence of liver disease

**DOI:** 10.1093/rap/rkae117.052

**Published:** 2024-11-05

**Authors:** Marvi Iftikhar, Alan Steur

**Affiliations:** Rheumatology, Wexham Park Hospital, Slough, United Kingdom; Rheumatology, Wexham Park Hospital, Slough, United Kingdom

## Abstract

**Introduction:**

Viral infections can present with features suggestive of rheumatic disorders. Hepatitis B virus infection has been associated with various autoimmune phenomena. We present a case of a patient with a recent diagnosis of Sjögren’s syndrome (SS) presenting with cryoglobulinaemic purpura secondary to chronic hepatitis B virus infection in the absence of any features of liver disease. Both the SS and cryoglobulinemia were likely secondary to extra-hepatic hepatitis B virus infection. Prompt anti-viral treatment resulted in suppression of viral load and resolution of the rash with no recurrence over 12 month follow-up. We discuss this rare manifestation of hepatitis B infection.

**Case description:**

A 73-year-old lady of Jordanian origin with a recent diagnosis of SS was referred to our department for a second opinion after developing a purpuric rash with no other symptoms of multi-system disease. She described typical sicca symptoms and was using standard lubricants with good symptom control. Examination revealed a widespread purpuric rash predominantly of the lower limbs. Routine blood tests showed a normal biochemical profile including normal liver function, a normal ESR (13 mm/hr), CRP (2.8 mg/L) and total immunoglobulin levels (IgG 9.4, IgM 1.45, IgA 1.36 g/L).

An autoantibody profile confirmed high titre ANA (1:640), a positive ENA Anti-Ro antibody and a strongly positive RF (>200 IU/mL) with negative anti-CCP (<0.5 IU/mL). C3 was normal but C4 was suppressed (<0.03 g/L). A cryoglobulinemia screen was positive with an IgM kappa paraprotein. She was hepatitis B surface antigen positive, with positive hepatitis Be antibody and hepatitis B core total antibody with no IgM. Her viral load was 52 IU/ml. She was hepatitis C and HIV negative. She had no evidence of renal disease and nerve conduction studies were normal.

Intriguingly, there was no clinical evidence of acute or chronic liver disease and her liver function tests, AFP, liver ultrasound, and fibroscan were normal. Although her viral load did not meet the threshold for anti-viral therapy in patients with liver disease, it was deemed appropriate to commence treatment with entecavir for cryoglobulinaemic vasculitis (CV). At follow-up after 3 months her rash had resolved with an undetectable viral load. At nine months she has had no recurrence of the rash and a persistently undetectable viral load.

**Discussion:**

Cryoglobulins may cause injury to vessels and end-organs, resulting in skin changes, arthralgia, glomerulonephritis, and neuropathy. Type II/III are usually associated with autoimmune diseases, malignancy, or infection – typically hepatitis C, although 0.5-5.5% may occur due to hepatitis B. The incidence of hepatitis B virus-associated cryoglobulinaemic vasculitis (HBV-CV) specifically is unknown. Hepatitis B is a hepatotropic virus and will cause cirrhosis in the majority, but is also a lymphotropic virus and can cause extrahepatic manifestations in 20% of patients. Patients with HBV-CV demonstrated hepatitis in 80% and cirrhosis in 20%. Cases of HBV-CV without hepatic involvement are very rare.

One suggested classification of HBV-CV divides patients into mild-moderate (purpura, asthenia, arthralgia), severe (glomerulonephritis, necrotising skin ulcers, mononeuritis multiplex), and life threatening (progressive glomerulonephritis, GI vasculitis, acute hyper-viscosity, haemorrhagic alveolitis, CNS vasculitis).

Anti-viral monotherapy with nucleoside analogues (NA) has been advocated in mild-moderate HBV-CV. Studies show improvement in symptoms related to treatment-induced viral response. The aim is to eradicate or suppress the virus and NA have been shown to reduce viral replication in 90-100% of cases. In most cases, NA lead to suppression but not eradication of the virus, and patients usually require lifelong therapy. For more severe disease, antivirals plus immunosuppressants are advocated, including steroids, rituximab and plasma exchange.

Autoimmune disorders have been previously reported in patients with hepatitis B virus infection. SS has been described in up to 28% of cases of HBV-CV and Raynaud’s phenomena in up to 14%. However, understandably, the number of patients in these studies is small (18 patients) due to the rarity of HBV-CV overall. With NA treatment, resolution of SS was seen in 11% of patients. RF decreased with treatment but C4 tends to remain low, which was replicated in our case study. A third of patients show resolution of cryoglobulins.

**Key learning points:**

• This case highlights that chronic hepatitis B infection can occur in the absence of any hepatic involvement with normal liver function tests.

• HBV-CV is a rare manifestation of hepatitis B virus infection.

• In patients with HBV-CV, up to 20% have sicca syndrome and Raynaud’s phenomenon

• HBV-CV can cause asthenia, purpura and arthralgia as mild manifestations.

• In all patients with autoimmune disorders, one should consider hepatitis B and C testing - particularly with at-risk populations.

• HBV-CV should always be treated regardless of the severity of liver disease in order to prevent progression to more severe forms of the disease.

• Anti-viral treatment should be continued even after symptoms resolve. Symptom resolution is usually seen alongside viral suppression.

• Sicca symptoms may also resolve in this cohort with NA therapy.

• One should only consider stopping therapy in those with complete recovery; HBsAg loss and HBsAg seroconversion.

